# The moderating role of walking in the association between HDL cholesterol and cognitive function among Korean older adults: a nationwide population-based cross-sectional study

**DOI:** 10.3389/fpubh.2025.1637180

**Published:** 2025-08-20

**Authors:** Kyuyoung Cho, Hye Won Chai, Min Kyoung Park

**Affiliations:** ^1^Department of Child Development and Family Studies, Pusan National University, Busan, Republic of Korea; ^2^Research Institute of Human Ecology, Pusan National University, Busan, Republic of Korea; ^3^Institute for Engaged Aging, Clemson University, Seneca, SC, United States; ^4^Department of Psychology, Clemson University, Clemson, SC, United States; ^5^Office of Long Term Services and Supports, Maryland Department of Health, Baltimore, MD, United States

**Keywords:** biopsychosocial perspectives, cognitive function, HDL cholesterol, walking, Korean older adults

## Abstract

**Background:**

The biopsychosocial model of dementia emphasizes an integrative approach that takes into account the joint effects of biological and behavioral processes relevant to cognitive function. Based on this model, this study examined the interactive effects of biological (i.e., high-density lipoprotein; HDL) and behavioral (i.e., frequency of walking) factors on cognitive function (measured using KDSQ-C) among South Korean (hereafter “Korean”) older adults. We conducted a subgroup analysis to explore whether the interaction of these factors differs depending on older adults’ history of chronic conditions.

**Methods:**

This study used cross-sectional Korean National Health Insurance Service data from 2009 that included a sample of older adults who remained qualified for health insurance and medical aid (*n* = 20,162). Linear regression models that tested for the interaction effects between HDL and frequency of walking on cognition were analyzed separately for two groups – no history of hypertension or diabetes and those with a history of hypertension or diabetes.

**Results:**

Results showed significant interaction effects (*β* = −0.024, *p* < 0.05) between HDL cholesterol and walking only among older adults with a history of chronic diseases. Specifically, more frequent engagement in walking buffered the negative effect of low HDL cholesterol on cognitive function for those with a history of hypertension or diabetes.

**Conclusion:**

These findings show that regular walking significantly attenuates the negative impact of low HDL cholesterol on cognitive function among Korean older adults with a chronic disease history. This highlights the importance of developing tailored intervention programs that emphasize the health benefits of walking, particularly for older adults with hypertension and diabetes, to help mitigate cognitive decline and promote overall cognitive health.

## Introduction

Korea has been experiencing a dramatic increase in the number of older population, with the number of older adults aged 65 and older projected to increase from 17.5% in 2022 to 20.6% in 2025 (1). Declines in cognitive functioning are one of the major health concerns in aging societies including Korea, and prior research has identified various correlates of cognitive function including biological and health behavioral factors (2, 3). One of the widely-examined biological correlates of cognitive function is high-density lipoprotein (HDL) cholesterol, which is considered as a “good cholesterol” that absorbs excess cholesterol from the blood and transports it to the liver to remove it from the body (4). In addition to its anti-inflammation, pro-endothelial function that is protective of cardiovascular health (5), increasing number of studies also show that lower levels of HDL cholesterol is associated with cognitive decline and higher risk of cognitive impairment and Alzheimer’s Disease and Related Dementia (6–8). This could be attributed to the biological mechanism where lower level of HDL cholesterol is associated with higher cardiovascular risk, which in turn poses negative influence on cognitive function (9). As for health behavioral factors, physical activity is commonly explored, with results showing that lack of physical activity including walking is a significant risk factor of cognitive decline and dementia (10–14).

However, existing research that identified risk and protective factors of cognitive function often focus either on biological or behavioral factors. Given that cognition is influenced by a simultaneous interplay of multiple factors, examining biological and health behavioral factors separately limits a holistic understanding of cognitive function in later life. According to the biopsychosocial model of dementia (15, 16), taking an integrative approach that takes into account the joint effects of biological and behavioral processes relevant to cognitive function is important in having a more in-depth understanding of cognitive functioning, which could be used to better inform interventions that aim to protect cognitive decline in later life.

Among the diverse types of healthy behaviors, walking in particular can be protective against the detrimental effects of biological risk factors (e.g., inflammation, cardiometabolic conditions, cholesterol) on cognition by increasing older adults’ resilience to adverse biological consequences (17). Walking is a widely preferred and easily accessible form of physical activity for older adults, as it requires no special skills or equipment and can easily fit into their daily life (18). Engaging in frequent walking may reduce the negative effects of biological risk by activating brain plasticity, stimulating neurogenesis, reducing inflammation, and optimizing hormonal stress responsive systems (19). Indeed, several studies find that lack of physical activity exacerbates the biological risk of cognitive decline while engagement in physical activity buffers the negative effect of biological risk factors (20–22). However, these studies focus on inflammation and metabolic syndrome; whether and how physical activity, specifically the frequency of walking, modulates the association between HDL cholesterol and cognitive function remain to be explored.

In examining the biological and behavioral correlates of cognitive function, one important factor to consider is physical health. The biopsychosocial model of dementia posits that different factors and processes that are relevant to cognitive function are closely related to one another, which suggest that the role of biological and behavioral correlates and their interactions in cognitive function may be dependent on individuals’ physical health. In other words, the well-established associations of biological and behavioral factors (e.g., HDL cholesterol and physical activity) with cognitive function may differ depending on preexisting health conditions. Hypertension and diabetes may be the most relevant in this regard, as these two conditions have long been identified as strong risk factors of AD/ADRD and cognitive impairment (23, 24). While there are separate studies that examined the correlates of cognitive function among patients with hypertension (25) or diabetes (26) and among healthy individuals without any conditions (27), whether and how the dynamic interplay between biological and behavioral factors related to cognitive function differ between those with and without health conditions remain less understood. Prior studies on the biological and behavioral correlates of cognitive function often include chronic conditions as a covariate, but their role as a potential modifying factor remains under examined. Given that older adults with chronic conditions often have dysfunctional metabolism or exercise capacity, stratifying by chronic conditions and examining whether the cognitive benefits of walking in the presence of biological risk differ can offer insights for precision prevention. In addition, understanding such differences could be helpful in developing tailored interventions that are designed to protect older adults against cognitive decline based on their preexisting health conditions.

Therefore, using HDL cholesterol and frequency of walking as the measures of biological and behavioral factors, this study aimed to explore the interactive effect of biological and behavioral factors on cognitive function among Korean older adults. Specifically, we tested whether the association between HDL cholesterol and cognitive function differed by frequency of walking. We further examined whether the moderating role of walking differed between those with and without a history of diabetes or hypertension. Based on the previous literature, we hypothesized that lower HDL cholesterol will be associated with worse cognitive functioning, and that this association will be moderated by frequency of walking such that the association will be weaker among older adults with more frequent walking. We further hypothesized that the moderating effect of walking will differ by chronic condition history, where the buffering role of walking will be stronger for older adults with a history of diabetes and hypertension.

## Methods

### Data and sample

The National Health Insurance Service - Senior Database (NHIS-Senior; 2002~2019) offers a unique advantage for examining Korean older adults’ health. As a part of the country’s universal healthcare system, the NHIS-Senior covers almost all of the Korean population, thereby minimizing selection bias. These features support rigorous cross-sectional analysis of the interactions between biological and behavioral factors related to cognitive function, with sufficient power for subgroup analyses by chronic disease status.

Data for this study were drawn from the 2009 NHIS-Senior dataset of the National Health Insurance Service (NHIS; NHIS-2024-2-141), which includes Korean older adults aged 60 to 80 years who were eligible for National Health Insurance or Medical Aid in 2008. Approximately 511,953 individuals - representing 8% of about 6.4 million older adults in this age group - were surveyed regarding their socioeconomic background, history of hospital visits, results of medical check-ups, and usage of long-term care services (28).

**Figure 1:**
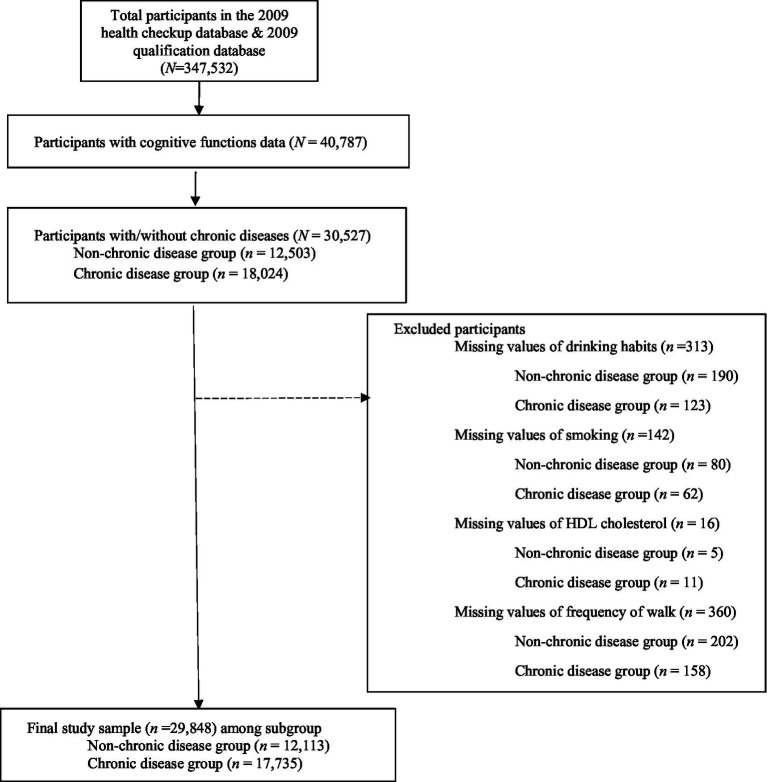
Flow chart of study participants.

Figure 1 presents the final sample selection process. We first merged the health check-up database and the qualification database that included study variables (*N* = 347,532). Because the national health screening guidelines primarily targeted individuals aged 66, 70, and 74 for cognitive screening, a large proportion of the initial sample who did not meet age requirements were missing on cognitive function data and thus were dropped from the analyses sample. Participants with a valid cognition function score was *N* = 40,787.

From this subset of participants, we further classified participants into two groups based on the presence or absence of chronic diseases (i.e., hypertension or diabetes): the non-chronic disease group (*n* = 12,503; does not have a history of diagnosis with either hypertension or diabetes) and chronic disease group (*n* = 18,024; has a history of diagnosis with hypertension or diabetes, or both). After excluding cases with missing values on key variables, the final analytic sample consisted of 29,848 individuals, including 12,113 in the non-chronic disease group and 17,735 in the chronic disease group.

We used a health check-up database to obtain data on HDL cholesterol, frequency of walking, cognitive function, drinking, smoking, and chronic diseases. The qualification database provided information on sex, age, and income quintile. The final analytic model included cognitive function as the dependent variable; HDL cholesterol and frequency of walking as independent variables; and sex, age, income quintile, drinking habits, and smoking as covariates – examined separately for groups with and without a history of chronic disease. Approval of research was obtained from the Institutional Review Board (IRB) of Pusan National University (PNU IRB/2023_156_HR).

### Measures

#### Cognitive function

Cognitive function was measured using the Korean Dementia Screening Questionnaire-Cognition (KDSQ-C). KDSQ-C is a subset of the larger KDSQ, and focuses on KDSQ items related to cognition. KDSQ is a validated questionnaire developed to screen early dementia patients in Korea. More information about the development of KDSQ can be found elsewhere (29). KDSQ is found to have high validity and reliability (*r* = 0.81). With KDSQ-C, we calculated the sum of five items, each ranging from 1 = *not at all* to 3 = *very much, where* higher scores indicate poorer cognitive function. Examples of items are: “Do you consider your memory to be lacking compared to your friends or colleagues?” and “Do you think it has become more difficult for you to perform everyday tasks?” Cronbach’s *α* for each group were *α* = 0.878 for the non-chronic disease group (no history of hypertension or diabetes) and *α* = 0.865 for the chronic disease group (has a history of hypertension or diabetes).

#### HDL cholesterol

HDL cholesterol was coded according to Panel NCEPNE (2002). HDL cholesterol was dichotomized as the following: <40 mg/dL for males and <50 mg/dL for females were coded as HDL risk factor (=1), and higher values as no-HDL risk factor (=0).

#### Frequency of walk

Frequency of walk was measured by asking how often respondents walked for more than 30 min during the past week. The question specifically asked, “During the past week, on how many days did you walk for a total of at least 30 min a day, combining walks of at least 10 min each time (for example, light exercise, commute, leisure walking)?” Responses ranged from 0 = *none* to 7 = 7 days a week.

#### Chronic condition

NHIS-Senior had two items that asked if respondents have been diagnosed with hypertension or diabetes. Based on the responses, this study divided the sample into two groups: the no-chronic diseases group (no history of hypertension or diabetes) and the chronic diseases group (has a history of being diagnosed with hypertension or diabetes).

#### Covariates

Covariates were sex (male = 0, female = 1), age (years), and income quintile (ranged from 0 to 10), smoking (not-smoking or smoked in the past = 0, currently smoking = 1). Drinking habits (ranged from 0 = never to 7 = 7 days a week).

### Plan of analyses

First, we conducted descriptive statistics and frequency analysis of the study sample. Second, linear regression models were analyzed with HDL cholesterol as the independent variable and cognitive function as the dependent variables. To test for the moderating effect of walking frequency, an interaction term between HDL cholesterol and walking frequency was added to the analyses model. The moderation analysis aimed to examine whether the relationship between HDL cholesterol and cognitive function differs by frequency of walk. All models were analyzed separately for two subgroups: the no-chronic disease group and the chronic disease group.

## Results

### Demographic characteristics

Table 1 shows the demographic characteristics of the sample for this study among the non-chronic disease group and chronic disease group. For the no-chronic disease group, 48.9% were male, the mean age was 68.50 (*SD* = 4.05), and the income quintile was 6.33 (*SD* = 3.10). The percentage of respondents with HDL cholesterol risk factor was 26.2%, frequency of walk was 2.55 (i.e., about 2.5 times a week; *SD* = 2.69), and the score for cognitive function was 6.51 (*SD* = 2.09).

**Table 1:** Sample demographic characteristics.

Variables	N(%)/M(SD)	Categories	No-chronic disease group (*n* = 12,113)	Chronic disease group (*n* = 17,735)	T-test /Chi-square test
Sociodemographic factors					
Sex (male = 0, female = 1)	*N* (%)	Male	5,923 (48.9)	7,672 (43.3)	−9.646***
		Female	6,187 (51.1)	10,063 (56.7)	
Age (years)	*M* (*SD*)		68.50 (4.05)	69.20 (3.39)	−15.298***
Income quintile (1 to 10)	*M* (*SD*)		6.33 (3.10)	6.44 (3.12)	−3.027**
Drinking habits (0 to 7)			0.83 (1.72)	0.76 (1.64)	3.679***
Smoking (current = 1)	*N* (%)	Currently not smoking	10,237 (84.5)	15,736 (88.7)	10.396***
		Currently smoking	1,876 (15.5)	1,999 (11.3)	
Biological factor					
HDL cholesterol (risk factor = 1)	*N* (%)	No risk	8,941 (73.8)	11,985 (67.6)	−11.718***
		At risk	3,172 (26.2)	5,750 (32.4)	
Behavioral factor					
Frequency of walk (0 to 7)	*M* (*SD*)		2.55 (2.69)	2.84 (2.73)	−9.177***
Cognitive function (score, 1–15)	*M* (*SD*)		6.51 (2.09)	6.50 (2.03)	0.340
Number of Chronic diseases					
One (hypertension or diabetes)	*N* (%)		-	14,381 (81.1)	
Two (hypertension and diabetes)	*N* (%)		–	3,354 (18.9)	

**p < 0.01; ***p < 0.001.

For the chronic disease group, 43.3% were male, mean age was 69.20 (*SD* = 3.39), and income quintile was 6.44 (*SD* = 3.12). The percentage of respondents with HDL cholesterol risk factor was 32.4%, frequency of walk was 2.84 (i.e., about 2.8 times a week; *SD* = 2.73), and the score for cognitive function was 6.50 (*SD* = 2.03). The differences between the two groups were significant for all variables except cognitive function.

### The moderating effect of walking frequency in the association between HDL cholesterol risk and cognitive function

Table 2 shows the moderating effect of walking frequency on the association between HDL cholesterol and cognitive function, after adjusting for all covariates. For the no-chronic disease group, HDL cholesterol was not significantly associated with cognitive function. However, walking frequency was significantly associated with cognitive function (*β* = −0.016, *p* < 0.05, 95% CI = [−0.03, −0.0004]), where one more day of walking during the past week was associated with better cognitive function by 0.016 points. The interaction between HDL cholesterol and walking was not significant, which indicates that the association between HDL cholesterol and cognitive function did not differ by the frequency of walk among the no-chronic disease group.

**Table 2:** Linear regression results on the associations between HDL cholesterol and cognition and the moderating effect of walk frequency, by chronic disease history.

Variables	No-chronic disease group (*n* = 12,113)			Chronic disease group (*n* = 17,735)		
	*B*	*SE*	*β*	*B*	*SE*	*β*
Constant	3.929***	0.322		6.078***	0.314	
Covariates						
Sex (male = 0, female = 1)	0.341***	0.044	0.082	0.358***	0.036	0.087
Age (years)	0.034***	0.005	0.066	0.002	0.005	0.003
Income quintile (1 to 10)	0.006	0.006	0.009	0.010*	0.005	0.015
Drinking habits (0 to 7)	0.071***	0.012	0.059	0.026	0.010	0.021
Smoking (current = 1)	0.004	0.057	0.001	0.058	0.051	0.009
Biological factor						
HDL cholesterol (risk factor = 1)	0.071	0.059	0.015	0.138**	0.047	0.032
Behavioral factor						
Frequency of walk (0 to 7)	−0.016*	0.008	−0.021	−0.006	0.007	−0.008
Interaction term						
HDL cholesterol x Frequency of walk	0.016	0.016	0.013	−0.024*	0.012	−0.023

For the outcome variable, higher values indicate worse cognitive function.

B = Unstandardized beta coefficient; SE = Standard error.

*p < 0.05; **p < 0.01; ***p < 0.001.

For the chronic disease group, results showed that HDL cholesterol was significantly associated with cognitive function (*β* = 0.138, *p* < 0.01, 95% CI = [0.05, 0.23]), where having HDL cholesterol risk factor (i.e., low levels of HDL cholesterol) was associated with lower cognitive function by 0.138 points compared to not having the HDL cholesterol risk factor. Frequency of walking was not significantly associated with cognitive function. The interaction between HDL cholesterol and Walking frequency was significant for the chronic disease group (*β* = −0.024, *p* < 0.05, 95% CI = [(−0.0471, −0.0002)]), which indicates that the association between HDL cholesterol and cognitive function differed by the frequency of walk for this group.

**Figure 2:**
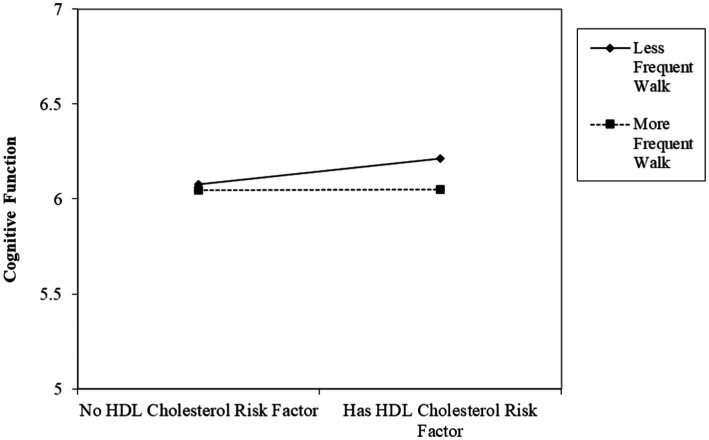
The associations between HDL cholesterol and cognitive function by the frequency of walking among Korean older adults with a chronic disease history. (Higher cognitive function score indicates worse cognition).

The interaction effects are plotted in Figure 2. More frequent walk was drawn at one standard deviation above the sample mean and less frequent walk was drawn at one standard deviation below the sample mean. Figure 2 suggests that the positive association between HDL cholesterol risk factor and cognitive function was weaker among older adults with a history of chronic condition who walk more frequently (dotted line) compared to those who walk less frequently (solid line), as indicated by the flatter dotted line. For example, estimates based on the regression coefficients presented in Table 2 show that for older adults with a history of chronic condition who do not engage in any walking, having a HDL cholesterol risk factor is associated with worse cognitive function by 0.138 points. However, for older adults who walk for more than 30 min 3 days a week, having a HDL cholesterol risk factor is associated with worse cognitive function by 0.066 points (0.138–0.024*3 = 0.066). Simple slope tests that examined the association between HDL cholesterol risk factor and cognition at low, medium, and high levels of walking frequency among older adults with chronic disease history showed that at low levels of walking frequency, having HDL cholesterol risk factor was marginally associated with worse cognition (*β* = 0.112, *p* = 0.054). The association was not significant at medium and high levels of walking frequency.

## Discussion

Using a nationwide population-based data collected from Korean older adults, this study examined the moderating role of walking frequency in the association between HDL cholesterol risk factor and cognitive function and whether these associations differ depending on the history of chronic conditions such as hypertension and diabetes. Results showed that among older adults with chronic conditions, engaging in frequent walking was protective of the negative effect of low HDL cholesterol on cognition. The interaction between HDL cholesterol risk and frequency of walking was not significant for those without a history of chronic conditions.

Based on the two separate lines of literature that identifies biological and behavioral factors related to cognitive functions in later life, prior studies consistently have examined the protective role of healthy behaviors against the biological risk factors of cognitive impairment. However, the dynamics involving HDL cholesterol, a biological factor that began to gain attention as a significant correlate of cognitive function (6), remain less understood. Also, the extent to which such relationships differ depending on the history of chronic conditions (e.g., hypertension, diabetes), which in and of itself is significantly related to cognitive impairment, has not yet been fully explored. Therefore, based on the biopsychosocial model of dementia (15, 16), this study contributes to the line of research on multidimensional correlates of cognition by emphasizing the interplay between biological (i.e., HDL cholesterol), behavioral (i.e., walking), and physical (i.e., chronic conditions) factors related to cognitive function in older adults.

Our results showed that engaging in frequent walking is protective of the detrimental effects of low HDL cholesterol on cognition only for older adults with a history of hypertension and diabetes, and not for those without a history of chronic condition. This is in line with findings from a previous study that compared frail and non-frail older adults (30) which found that physical exercise was particularly more beneficial for cognitive function for frail older adults compared to non-frail older adults. In addition, findings from a meta-analysis (31) show that physical activities play a significant role in managing chronic conditions and enhancing cognitive function. For patients with diabetes in particular, engaging in physical activities was crucial in regulating blood glucose levels which can influence cognitive functioning in the long run. Given the importance of physical activities for older adults with chronic conditions, these findings, along with the findings from our study, suggest that engaging in frequent walking may also matter more for the cognitive functioning of older adults with a history of hypertension or diabetes compared to those without any conditions. Frequent walking might be an important factor in maintaining and improving cognitive function among older adults with chronic conditions, by mitigating the negative consequences of having higher biological risk (i.e., low HDL cholesterol).

Among older adults with a history of hypertension or diabetes, our findings showed that engaging in frequent walks buffered the negative effect of low HDL cholesterol on cognitive status. It is possible that walking involves biological and psychological benefits of physical activity that protects the brain against the risks of low HDL cholesterol. Biologically, physical activity enhances blood flow and vascular health that improve cerebral perfusion and enable the brain to receive more oxygen (32, 33), which may strengthen the brain to be more resilient against the effect of risk factors. Engaging in physical activity is also known to reduce inflammation and oxidative stress (34, 35), which can contribute to enhanced brain functioning that buffers the biological risk posed by low HDL cholesterol. Also, studies find that physical activity stimulates the production of brain-derived neurotrophic factor (BDNF) that supports neuroplasticity (36, 37), which in turn, can promote cognitive function and reduce the negative impact of HDL dysfunction on the brain.

Engaging in physical activity such as walking may also protect the brain from the detrimental effects of low HDL cholesterol by enhancing psychological well-being (38–40). For example, physical activity reduces depressive symptoms and increases positive mood by modulating the peripheral levels of BDNF and decreasing oxidative stress (41, 42). These physiologic mechanisms, in turn, can improve brain health and mitigate the biological risk posed by low HDL cholesterol. Also, studies find that engaging in physical activity helps reduce unhealthy behaviors such as smoking and drinking that are detrimental to cognitive functioning (38, 43). In this regard, physical activity may buffer the impact of biological risk factors by decreasing unhealthy behaviors and increasing healthy behaviors.

There are several limitations to note. First, cognitive function in our data was collected from a limited number of participants, mainly from those who were aged 66, 70, and 74 years old at the time of the data collection. Also, information on participants’ education levels was not available in our data. Given that level of education is a significant correlate of cognitive functioning, the association between HDL cholesterol and cognitive function may in part be explained by including education. Second, this study only used frequency of walk to measure physical activity due to the limitations of the data. The moderating role of physical activity may differ depending on the type (e.g., aerobic exercise, weight training), intensity (e.g., light, moderate, intense), or exposure time of physical activities. Third, in measuring the frequency of walk, this study only measured the frequency of at least 30-min walk during the past week. Information on frequency of walking across a longer period of time (e.g., across multiple weeks or during a month) was not measured, which may limit the accurate measurement of our sample’s engagement in walking activities. Also, frequency of walk was a self-reported variable, which may be subject to recall bias or social desirability bias. As such, the actual engagement in walking activities might be different from the reported values, potentially introducing measurement error into the analysis. Fourth, while this study adjusted for multiple covariates that may be associated with cognitive function, residual confounding may remain due to unmeasured factors such as psychological factors (e.g., depressive symptoms), social factors (e.g., family relationships), undiagnosed health conditions, or lifestyle variables (e.g., sleep quality, diet, and stress). Based on the biopsychosocial model of cognitive functions, future studies should also consider the role of contextual factors such as socioeconomic status that may influence the association between biopsychological factors and cognitive functioning. Fifth, this study did not include neuroimaging or biomarker data to objectively measure cognitive status, which may limit the precision of cognitive assessments. Sixth, although statistically significant, the effect size of the interaction term between HDL cholesterol risk factor and frequency of walk was small for this study (Cohen’s f^2^ = 0.001, which corresponds to the change in R^2^ between the regression models without and with the interaction term). This might be attributed to the measurement of walking frequency in our data that reflects engagement in walking activities within a short time window (i.e., during the past week). Future studies that examine the cumulative benefits of walking across a longer period (e.g., months or years) may find larger effect sizes. In addition, given the cross-sectional nature of the study design, causality cannot be inferred from the study findings. It is possible that older adults with worse cognitive function engage in less frequent walking. Future studies should utilize longitudinal data to better examine the causal relationships between health behaviors and cognition. Lastly, this study focused on the history of hypertension and diabetes, which are two of the most prevalent chronic conditions among Korean adults. Future studies should expand upon this research and consider other types of chronic conditions.

Despite the limitations, there are important strengths to our study design. First, we focused on regular walking as a potential buffer against the negative effects of biological risk on cognition. Walking is a cost-effective and accessible health behavior that older adults can easily practice in their daily lives, which can be an effective point of lifestyle intervention. Second, we examined differences by history of chronic health conditions, which is an easily obtainable information from medical check-ups. This can be used to design tailored interventions for diverse subgroups of older adults based on their medical history. Third, we used data from NHIS-Senior, which was collected from a nationally representative sample of Korean older adults. This represents the value of leveraging comprehensive national health data for aging research. Using the NHIS-Senior dataset allows to capture health profiles of a large, representative sample of Korean older adults, which strengthens the utility of the findings for public health policy and intervention.

In addition, findings from this study have potential practical implications for both clinical and public health settings. Given that having a HDL cholesterol risk factor was associated with worse cognitive health only among older adults who walked less frequently compared to those who walked more frequently, clinicians could incorporate physical activity counseling as part of preventive strategies for cognitive health for at-risk older populations. Public health professionals may also consider developing community-based walking programs targeting older adults with biological and physical risk profiles as a cost-effective intervention to preserve cognitive function. As the burden of cognition-related diseases continues to grow (44), using rich administrative health data such as the one used in our study will be critical in informing scalable public health interventions.

In conclusion, this study highlights the importance of considering the history of chronic conditions such as hypertension and diabetes, in identifying behavioral factors that may buffer against the negative effects of biological risk factors (i.e., low HDL cholesterol) on cognitive function among older adults. Expanding upon the findings of this study, future research should consider incorporating objective measures of physical activity (e.g., accelerometers) and cognitive function (e.g., neuroimaging or task-based assessments) in study design. Studies can also examine the role of other lifestyle factors—such as sleep quality, diet, and stress—as mechanisms or moderators of biomarker-cognition relationships. Longitudinal or intervention studies are also needed to explore the causal relationships and to evaluate the effectiveness of walking-based interventions in maintaining cognitive health among older adults with biological and physical risk factors.

## Data Availability

The data analyzed in this study is subject to the following licenses/restrictions: access to data is restricted and only available through permission and approval by the Korean National Health Insurance System (NHIS). Requests to access these datasets should be directed to https://nhiss.nhis.or.kr/. This study used NHIS-NSC data and approval number for this study is: NHIS-2024-2-141. The content of this study is solely the responsibility of the authors and does not represent the official views of the NHIS.
